# Broadband Tunable Infrared Light Emission from Metal-Oxide-Semiconductor Tunnel Junctions in Silicon Photonics

**DOI:** 10.1021/acs.nanolett.3c03684

**Published:** 2023-12-05

**Authors:** Michael Doderer, Killian Keller, Joel Winiger, Michael Baumann, Andreas Messner, David Moor, Daniel Chelladurai, Yuriy Fedoryshyn, Juerg Leuthold, Jared Strait, Amit Agrawal, Henri J. Lezec, Christian Haffner

**Affiliations:** † Institute of Electromagnetic Fields (IEF), ETH Zurich, 8092 Zurich, Switzerland; ‡ Physical Measurement Laboratory, National Institute of Standards and Technology, Gaithersburg, Maryland 20899, United States; § Interuniversity Microelectronics Centre (imec), Remisebosweg 1, 3001 Leuven, Belgium

**Keywords:** Light emission, Tunneling, Photonics, Cavity enhancement, Silicon

## Abstract

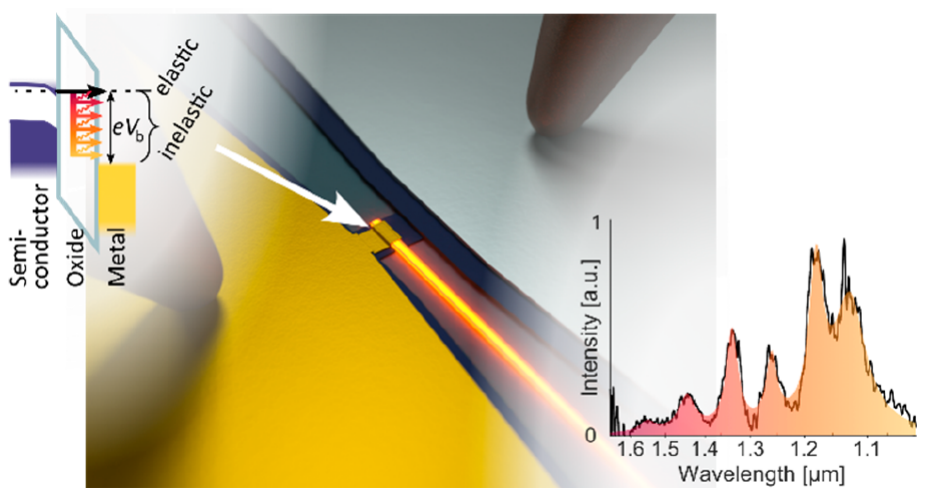

Broadband near-infrared light emitting tunnel junctions are demonstrated with efficient coupling to a silicon photonic waveguide. The metal oxide semiconductor devices show long hybrid photonic–plasmonic mode propagation lengths of approximately 10 μm and thus can be integrated into an overcoupled resonant cavity with quality factor *Q* ≈ 49, allowing for tens of picowatt near-infrared light emission coupled directly into a waveguide. The electron inelastic tunneling transition rate and the cavity mode density are modeled, and the transverse magnetic (TM) hybrid mode excitation rate is derived. The results coincide well with polarization resolved experiments. Additionally, current-stressed devices are shown to emit unpolarized light due to radiative recombination inside the silicon electrode.

Light emission from electron tunnel junctions is a fascinating mechanism for studying light–matter interaction. Historically, the process has been first described in metal–insulator–metal junctions (MIM),^1^ where the emission stems from the tunneling of electrons through a few nanometer thin oxide barrier. A fraction of the tunneling electrons will tunnel inelastically and primarily excite highly confined gap-plasmon–polariton modes. After these strongly confined and lossy plasmons are excited, they are then usually either scattered out or radiated out by plasmonic antennas, leading to far field photon emission.^2,3^ The emitted light can be used, for example, as an analytic tool in inelastic electron tunneling spectroscopy.^4^ In the world of superconductors, the same principle of inelastically tunneling electrons is used to excite microwave photons in Josephson junctions, where not just spontaneous but also stimulated emission has been shown.^5^ In general, light emitting tunnel junctions (LETJ) do not require the integration of a direct bandgap material, which has sparked interest in their usages as substrate-independent integrated light sources. Additionally, the tunneling process itself happens on attosecond time scales,^6^ which should allow for extremely fast modulation of such light sources that would presumably only be limited by their resistance-capacitance (RC) time constants.

Since the first demonstration in 1976 of such inelastic tunneling light emission in MIM tunnel junctions,^1^ research has spread out to studies in scanning tunneling microscopes^7−10^ (STM), metallic nanophotonic geometries,^11−14^ as well as metal oxide semiconductor (MOS) tunnel junctions.^15−19^ Internal quantum efficiencies (emitted inelastic vs total tunneling rate) of ∼32% have been achieved by utilizing the large local optical density of states (LDOS) inherent to highly confined gap plasmons and an inelastic transition-tailored multiquantum-well structure.^20^ In other work, the significance of conserving the tunneling electrons’ momentum has been nicely demonstrated with graphene electrodes.^21,22^ And since tunnel junctions generate many hot electrons, discussions about all the possible plasmon emission mechanisms are still ongoing.^13,23^

Challenges with the MIM geometry are the high optical losses as well as the extreme confinement, with the latter making any coupling to photonic modes difficult. By transitioning to MOS structures, the coupling of the emitted light to a waveguide (WG) can be more easily addressed. Such MOS LETJ have already been demonstrated to emit visible light.^18,19^ However, these previous studies are performed close to the electrodes’ plasmonic resonances at visible light frequencies, and the large wave vector of the subdiffraction limited, lossy plasmons provides a challenge to couple the source to diffraction limited but lossless integrated photonic WGs for on-chip applications.

In this Letter, we demonstrate, to the best of our knowledge, a near-infrared (NIR) LETJ that for the first time couples light directly to an integrated silicon photonic circuit. Inelastically tunneling electrons in a metal-oxide-semiconductor tunnel junction are employed to excite light directly into a photonic–plasmonic hybrid WG mode. These hybrid plasmonic modes benefit from lower losses compared to gap-plasmonic modes (*l*_*prop*,MOS_ ≈ 10 μm vs *l*_*prop*,MIM_ ≈ 0.5 μm for Au-SiO_2_-Si and Au-SiO_2_-Au stacks, respectively, simulated at λ = 1500 nm). This allows for building of larger LETJ cavities that feature designed resonances with internal *Q*_MOS_ ≈ 150 that compares favorably to the traditional MIM structures with *Q*_MIM_ ≈ 17 for MIM LETJ. Due to the higher *Q*-factor, an MOS LETJ can feature narrower spectral line widths. However, the low propagation loss of a hybrid mode goes hand in hand with lower field confinement compared to gap-plasmon modes (LDOS: ρ_MOS_/ρ_0_ ≈ 300 vs ρ_MIM_/ρ_0_ ≈ 12000). The lower LDOS results in a smaller light emission rate per area, yet the low propagation loss allows for an effectively larger tunneling junction area, resulting in similar collected power levels of ∼20 pW, compared to an MIM LETJ with far field emission powers around ∼10 pW.^14^ Furthermore, the generated light features wave momentums that can be easily matched to low-loss photonic modes of integrated circuits as demonstrated here, with extraction efficiencies of 74_–8_^+6^%, as measured via photonic cutback measurements, where the uncertainty is given as the 95% confidence interval of the linear fit to the propagation losses (details in the Supporting Information).

The demonstrated MOS device was integrated into a silicon photonic circuit; see Figure 1a. The photonic circuit is composed of *h* = 340 nm thick silicon WGs, homogeneously doped with phosphorus with nominal doping density, *n*_phosphorus_ ≈ (5 × 10^18^) cm^–3^, on a silicon dioxide substrate. After patterning the silicon, micrometer-scaled MOS tunnel junctions were realized by sequential deposition of an approximately 2 nm thick silicon dioxide tunnel barrier via plasma enhanced atomic layer deposition (PE-ALD) and e-beam evaporation of an upper ∼100 nm thick gold electrode (further details in the Supporting Information), forming an optical Fabry–Pérot cavity.

**Figure 1:**
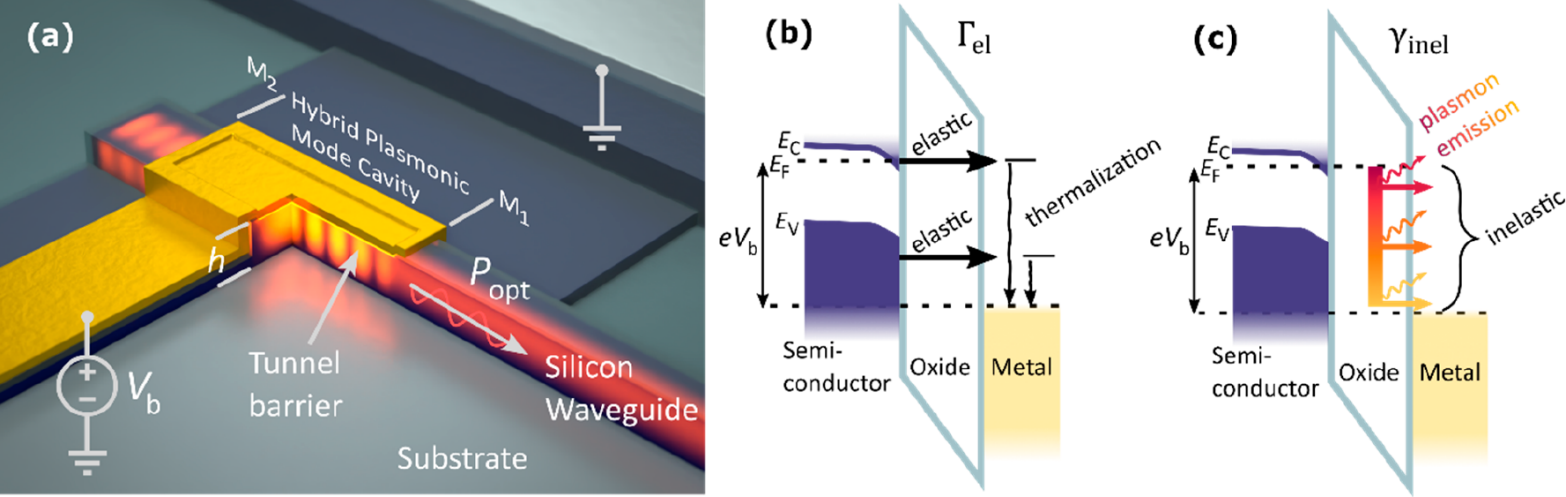
(a) Artistic illustration of the LETJ inside the hybrid plasmonic cavity coupling the emitted light out into a photonic WG. (b, c) Illustration of the electrodes’ band alignment and (b) elastic and (c) inelastic tunneling process.

Upon application of a bias to the tunnel device via electronic contacts to the gold and the doped silicon, a tunnel current can flow through the thin oxide. The majority of tunneling electrons will transition elastically from the silicon to the positively biased gold electrode, as illustrated in Figure 1b. These electrons are primarily thermalizing via nonradiative phonon–electron and electron–electron interactions to the Fermi level.^24^ In analogy to conventional solid-state light sources, electrons in the silicon electrode’s conduction band are in an excited state, and they can also relax to any free ground state in the positively biased gold electrode via an inelastic, radiative tunneling event, as illustrated in Figure 1c. In these radiative transitions, the electrons act as dipole-like sources with an electric dipole moment parallel to the tunneling direction, exciting primarily plasmonic modes.

To obtain a deeper understanding of the performance of the MOS LETJ and how silicon’s bandgap influences the tunneling transition probabilities and the resulting emission spectrum, we calculated the vacuum source spectrum (η_source_). This spectrally resolved internal quantum efficiency is modeled by calculating the elastic electron tunneling rate (Γ_el_) as well as the inelastic transition rate (γ_inel_) via Fermi’s golden rule^11,25^ (details are in the Supporting Information). Note that a dipolar interaction in a vacuum is assumed in order to deconvolute the effects of the electronic and optical density of states. The vacuum source spectrum is normalized by the much larger elastic electron tunneling rate Γ_el_, thus yielding the internal spectral quantum efficiency
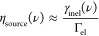
where the frequency of the emitted photons is ν. Both elastic and inelastic rates are modeled by considering tunneling probabilities and the electronic density of states (eDOS). To model the electron tunneling transition, we consider the two electrodes as weakly coupled and separated by a rectangular potential barrier.^11,26,27^ The finite height of the barrier results in a nonzero overlap of the exponential tails of the electrons’ wave functions in the silicon and gold electrode and subsequently allows the different tunneling transitions to occur.

Figure 2a shows the calculated vacuum source spectrum of the MOS system. As expected for single electron transitions, the emission follows the cutoff condition *hν* ≤ *eV*_b_, as the maximal emission energy cannot be larger than the applied bias over the tunnel barrier. Contrary to a MIM LETJ, MOS junctions can utilize a semiconductor’s bandgap to suppress elastic tunnel rates since for low applied biases only electrons from the accumulation layer at the interface can tunnel. For silicon, biases below 1.2 V thus suppress elastic tunneling from silicon’s valence band to the metal. This leads to a theoretical 1 order of magnitude increase in the source efficiency when emitting low energy photons. Experimentally, however, the InGaAs photodiode detectors used limit the optical detection bandwidth from approximately 1.1 to 1.65 μm, meaning we can only capture sufficient light emission for a bias range of 1.25 to 2.5 V, where the vacuum source spectrum is very broadband and relatively featureless, i.e., it does not change much with changing bias voltage. Figure 2b shows the integrated vacuum efficiency quantified as photons excited per tunneling electron. Low energy photons can be generated with more than a 10^–5^ vacuum efficiency (photon per electron, γ/*e*^–^, yield) as the elastic tunneling is suppressed. This compares very favorably to a MIM system, where we calculate a vacuum photon yield of only around 10^–7^ for a gold-oxide-gold tunnel junction using the same methodology.

**Figure 2:**
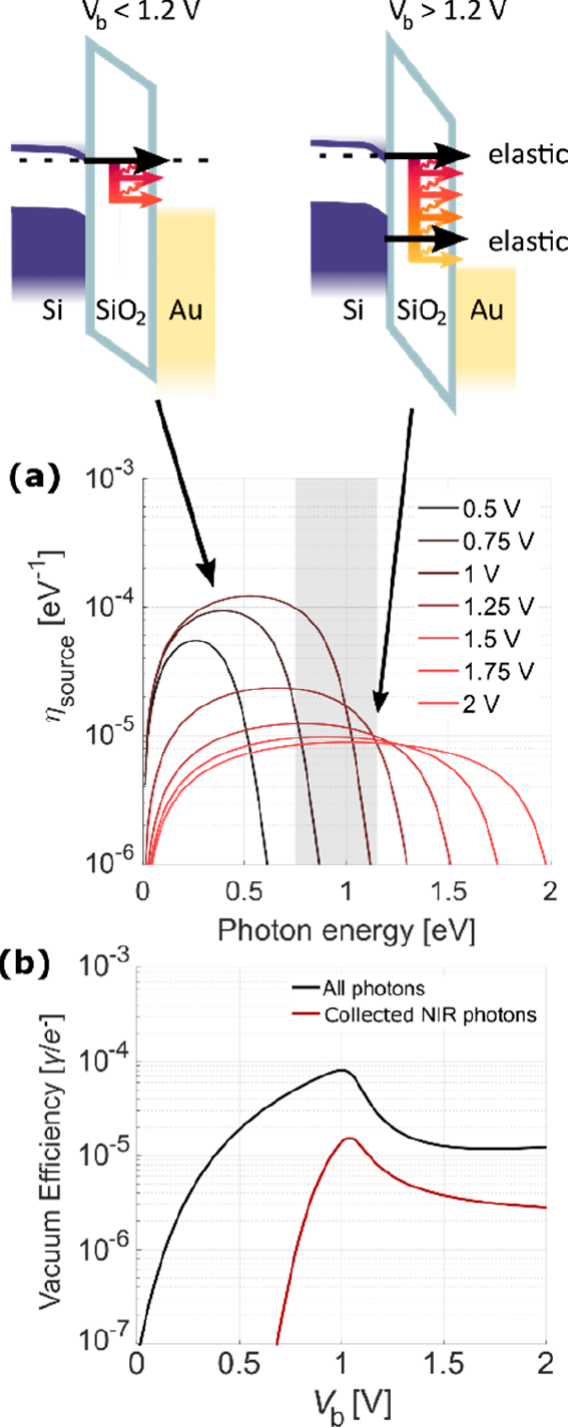
(a) Calculated vacuum source spectrum for the MOS junction, which disregards any effect of the optical environment (LDOS) on the inelastic transition rate. Shown above the plot are exemplary band diagrams for the below and above threshold applied biases. A 1 order of magnitude increased emission efficiency is visible when comparing the emission under 1 and 2 V bias. (b) Overall calculated vacuum efficiency for all emitted photons and for the collected NIR photons (λ_collected_ ≈ 1.1–1.65 μm).

The aforementioned inelastic electron tunneling rate is substantially enhanced by the LDOS of the optical mode within the WG structure (ρ_t_/ρ_0_, transverse confinement) and cavity (χ_cav_, longitudinal confinement). Assuming a dipolar transition, the dipole’s own emitted field is scattered back by material interfaces and drives or dampens its own oscillation,^28−31^ leading to a linear increase of γ_inel_ with the optical density of states ρ_opt_.^11^ For our device, we approximate the total normalized density of states by two contributions (details in the Supporting Information):
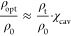
where ρ_0_ is the vacuum LDOS and ρ_opt_ is the total LDOS defined by the geometry. The enhancement of ρ_t_/ρ_0_ is due to the transverse confinement of the supported hybrid photonic–plasmonic mode, and the cavity factor (χ_cav_) only considers the longitudinal case of the hybrid mode inside the Fabry–Pérot cavity. For simplicity, we will first restrict our discussion to the case of the one-dimensional (1-D) heterogeneous layer stack (homogeneous along *x*); see Figure 3a.

**Figure 3:**
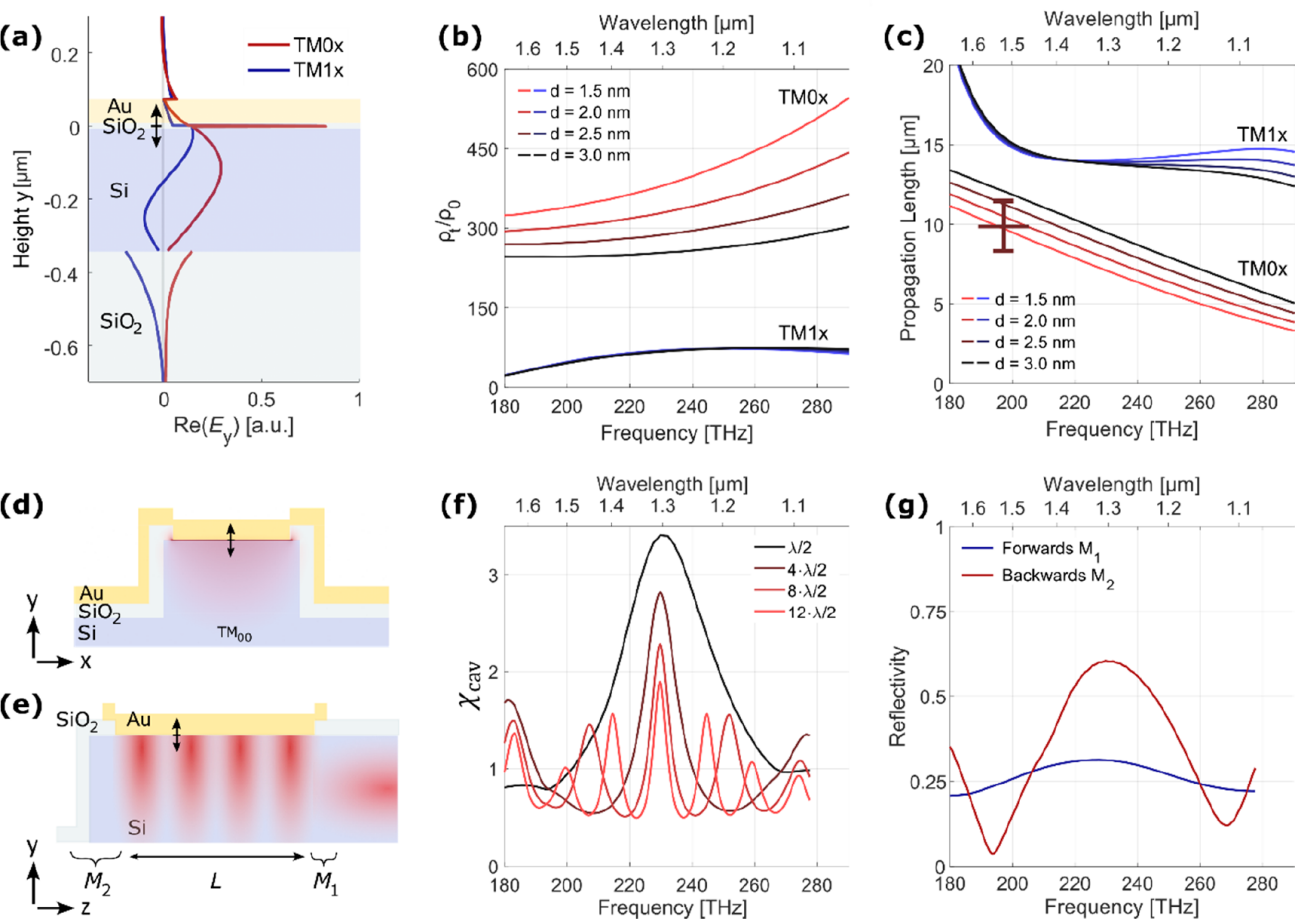
(a) MOS heterostructure shown together with the hybrid mode profiles TM_0*x*_ and TM_1*x*_, showcasing the strong enhancement inside the thin tunnel oxide layer, where a dipole is illustrated. (b) LDOS enhancement due to the transverse mode confinement. A dipole inside the tunnel barrier excites primarily the TM_0*x*_ mode over a wide frequency range. (c) For varying oxide thickness *d*, the hybrid plasmonic modes showcase low losses, which have been experimentally verified via cutback measurements (cross; the error bar signifies the 95% confidence interval of the fit to the experimental propagation losses for varying lengths). (d) Cross section through the WG with the dominant TM_00_ mode. (e) Longitudinal cross section through the hybrid cavity of length L with reflective mirrors M_1_ and M_2_ with the dominant TM_00_ mode. (f) Depending on the cavity length L, different out-coupled cavity enhancements are possible. (g) 3D finite-difference time-domain (FDTD) simulated reflectivities of the mirrors M_1_ and M_2_.

The mode nomenclature TM_*yx*_ is used with *y* (height) and *x* (width) representing the mode order number along the Cartesian coordinate system. The fundamental modes (TM_0*x*_) have a larger interaction with the dipole moment of the tunneling electrons compared to higher-order modes, which expresses itself by the larger calculated transverse LDOS; see Figure 3b. This calculation uses the angular spectrum representation, i.e., it considers all possible photon momenta emitted into the heterostructure, and specific modes, e.g., TM_0*x*_ are fitted. For our MOS stack, around 25% of the emitted NIR photons are directly emitted into the fundamental TM mode, while the rest are emitted into either free space or the substrate or end up in different modes (see the Supporting Information).

Higher-order WG modes in the lateral direction (*x* > 0) are suppressed by the narrow WG width (∼450 nm), and only the TM_00_ (shown in Figure 3d) and TM_10_ modes are supported for a wide frequency range. Suppressing the higher-order *x*-modes maximizes the emission/LDOS into a single mode due to the smaller mode volume (Supporting Information). If higher-order transverse modes, e.g., TM_01_, and TM_02_ are supported, the position-averaged dipole-like emission process will distribute its emitted power between the possible modes, effectively reducing the emission probability into the fundamental mode.

Confining the excited modes within a Fabry–Pérot cavity (see Figure 3e) results in a narrowing of the emission spectrum and gives rise to a discretization of the longitudinal mode density (i.e., enhancement of the LDOS, χ_cav_). Narrower emission spectra require a larger quality factor (*Q*_cav_) of the cavity, which is limited by either the intrinsic absorption losses or the coupling losses or both: 1/*Q*_cav_ = 1/*Q*_coupling_ + 1/*Q*_intrinsic_. We extract an intrinsic *Q*-factor of 136 ± 20 at a wavelength of 1500 nm from the measured propagation length of 9.6_–1.4_^+1.9^ μm, where the uncertainty comes from the 95% confidence interval of the fit to the experimental propagation losses. Both the simulation and measurement are shown in Figure 3c. Such *Q*-factors enable line widths of 10 nm and below, which is in strong contrast to the theoretical broad line width of 96 nm for a MIM LETJ emitting at 1500 nm.

For the effective LDOS enhancement of a cavity mode, where we assume homogeneously distributed dipoles along the cavity length,^28−30^ we estimate a 3.5× enhanced LDOS for a lambda-half-sized cavity; see Figure 3f. Shorter cavities provide a limited benefit to the LDOS due to increased coupling losses, as is evident by the broadening of the resonance, i.e., the emitted light is already overcoupled to the WG for our 2 μm long device and coupling losses dominate over propagation losses. We overcoupled the cavity to provide a feasible trade-off between spectral purity and extracted power. In detail, the cavity is terminated by a forward reflector (*M*_1_) based on a mode mismatch between the photonic and hybrid WGs. The backward reflector (*M*_2_) utilizes a resonant reflector formed by abrupt termination of a 500 nm long photonic section. The reflection coefficient of M2 is higher than that of M1, enabling emitted light to be coupled primarily to the WG; see Figure 3g.

To calculate the total emitted power spectral density, combining the vacuum source spectrum with the optical models, we get a scaling of
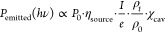
where *P*_0_ is the dipole emission spectrum in vacuum, *I* is the tunnel current, and *e* is the electron charge. The total simulated emission spectrum and its constituents are shown in Figure 4a. The experimental emission spectrum of a 2 μm long cavity, collected at the polished facet of an edge-coupled silicon photonic WG, is shown in Figure 4c. When comparing the simulated and measured spectra, we note an excellent agreement on most of the resonant peaks. The peaks were fitted with a sum of Lorentzians. Both center frequencies and *Q*-factors are well reproduced from our model, except for the center frequency of the highest frequency peak around 270 THz. The peak around 240 THz is significantly weaker, which we assume stems from a spatially uneven distribution of tunneling paths. Such spatially uneven tunneling rates are often observed in top-light emitting inelastic tunnel junctions.^2,19^ Position-dependent variations in the tunneling oxide thickness will significantly influence the tunneling rate and determine the excitation strengths of the various longitudinal cavity modes. Note that the differences between modes are limited to the peak power intensity, not the *Q*-factors, in our measurements.

**Figure 4:**
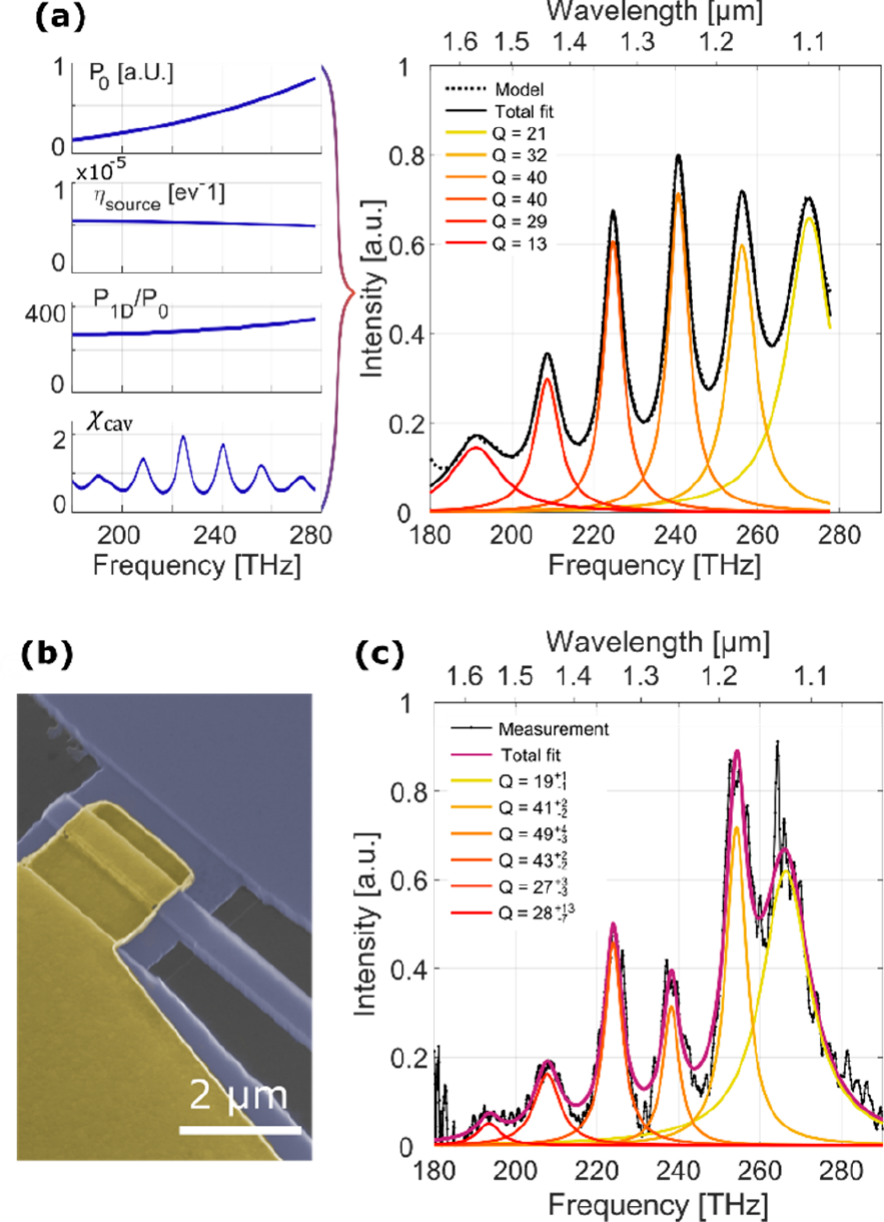
(a) Plots of calculated free space dipole emission, MOS vacuum source spectrum, transverse modal emission enhancement, and cavity enhancement. The simulated spectrum shows the dominance of longitudinal resonator modes due to χ_cav_. (b) Colorized SEM image of the single mode device. (c) TM emission spectrum of the 2 μm long device shown in (b), recorded with a source current density of 116 μA/μm^2^ through the area of the tunnel junction (*V*_*b*_ ≈ 2.45 V). The uncertainties in the *Q*-factor are the 95% confidence bounds of the multi-Lorentzian fit on the measurement.

In addition to the discussed inelastic tunnel emission, the combination of atomic-scale tunnel barriers and high fields can give rise to various other emission mechanisms in tunnel devices,^13^ such as hot electron emission, two-electron tunneling processes, photoluminescent centers in SiO_*x*_^32^ and radiative recombination in semiconductors. Some of these mechanisms can introduce emission of orthogonal polarized light (TE and TM), spectral shape broadening, or above-bias-threshold emission (*hν* > *eV*_b_).

In all of our devices, under sufficiently high bias, we measure not just TM polarized emission as expected but also some TE polarized emission, the strength of which varies strongly from device to device. The dipole moment of inelastic tunneling is perpendicular to the tunnel junction and thus should not excite TE polarized light that is parallel to the junction. However, in our devices under sufficiently high bias, electrons in the valence band of silicon are transmitted to the gold electrode, presumably primarily via localized defects in the tunnel barrier. With this, the many electrons in the accumulation layer at the oxide interface can readily recombine with the holes in the valence band, as shown in Figure 5a. A few of these recombination events interact with phonons and will emit unpolarized light, leading to measured TE and TM peaks around 270 THz. Figure 5b shows a polarization resolved emission spectrum for a 1 μm short, transverse, single-mode resonant device. The TM emission shows the expected broadband emission including cavity modes in addition to a broad peak around 270 THz. The same 270 THz emission is also the only one that is observed in the TE spectrum and resembles the previously reported luminescent recombination of carriers in silicon.^33,34^ The emitted light is broadened and red-shifted compared to the intrinsic band gap due to the strong band bending in the accumulation layer, where the majority of the recombination occurs.

**Figure 5:**
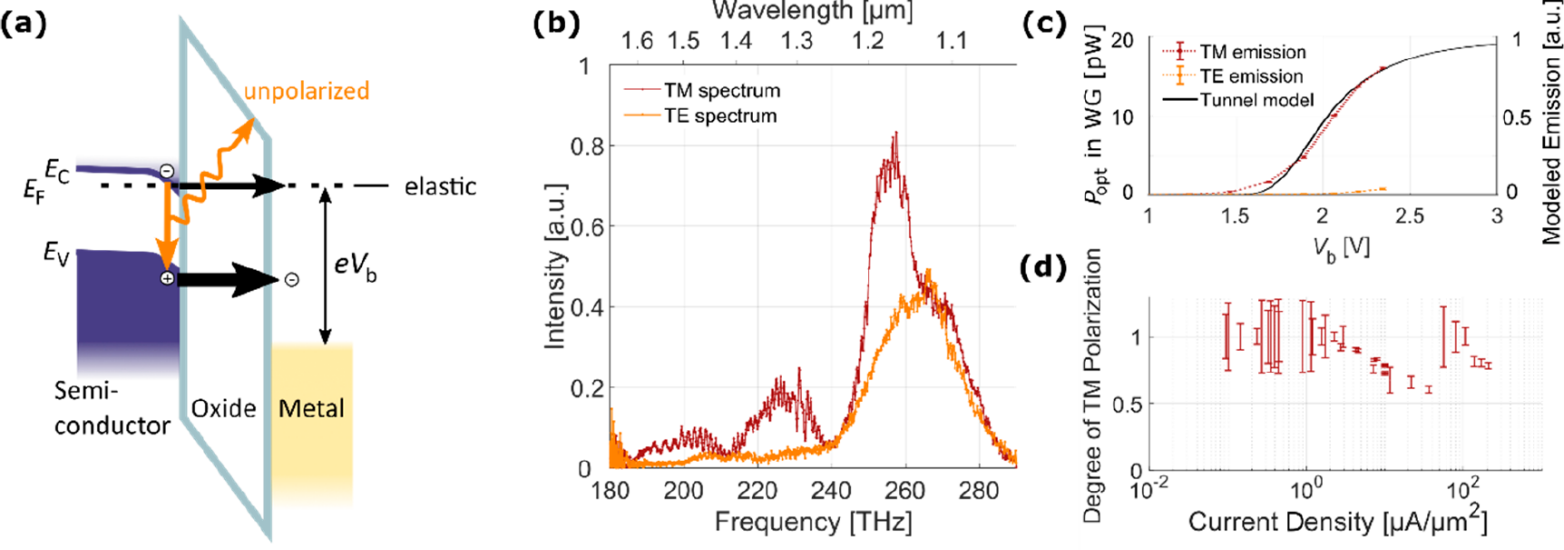
(a) Direct conduction of electrons from the silicon’s valence band to gold allows for recombination in the accumulation layer. (b) Polarization resolved emission spectrum showcasing the fundamentally different emission of TM and TE polarized light in a 1 μm long device. (c) For new, pristine devices, the TM emission clearly dominates and fits well to our inelastic tunneling model. Error bars signify one standard deviation of the measured background noise in the emission measurements. (d) Degree of polarization for the collected NIR frequencies for different devices operated with different current densities, where only current densities exceeding 25 μA/μm^2^ show partial TE emission. The error bars correspond to one standard deviation of the measured background noise.

While the total strength of this TE emission is strongly device-dependent, we can report that for pristine tunnel devices, usually no TE emission is recorded, with our largest measured polarization ratio being *P*_opt,TM_/*P*_opt,TE_ = 74. Note that this ratio is limited by the noise of the detector. For such devices, we measure a very good qualitative agreement between the TM emitted optical power and our inelastic tunneling model, as shown in Figure 5c. There, the collected TM and TE NIR emissions for varying applied voltages are shown, measured from a pristine, large-area (25 × 2 μm^2^) tunnel junction. Additionally, we calculate with our inelastic tunneling model the expected emitted power by integrating the inelastic transition rate over the collected emission frequencies and end up with a good match to the measured TM emission. However, for current-stressed devices, i.e., devices that have experienced sustained tunneling currents, the aforementioned TE emission can always be seen. Figure 5d shows the spread of different degrees of TM polarization, ζ_TM_ = *P*_opt,TM_/(*P*_opt,TM_ + *P*_opt,TE_) over a wide range of current densities of various tunnel junctions. We presume that with higher current stress the silicon dioxide tunnel barrier gradually degrades with a “soft” breakdown,^35,36^ which then enables higher conductance of valence band electrons and subsequent unpolarized emission by recombination. Based on these results, future experiments should take into account that not just one emission mechanism is at play in such tunnel devices, especially if semiconducting materials are involved.

For our MOS LETJ the overall photon emission efficiency, i.e., the probability for an electron to excite a guided hybrid plasmon at NIR frequencies that is also coupled into the photonic WG, is measured to be on the order of 10^–9^ to 10^–8^, where the large range is due to device variations between fabricated tunnel junctions. These MOS measured values are generally lower than values measured in different MIM tunnel junction systems and lower than the estimated NIR emission efficiency of approximately 4 × 10^–5^ from our model at 2 V. We assume that the quality of our tunnel junctions, deposited via plasma enhanced atomic layer deposition (PE-ALD), is not ideal and allows for further conductive channels through the oxide, increasing the total current through the device without light emission. Alternatively, our simplified tunnel rate model including a rectangular barrier without potential-barrier lowering might not be adequate to quantify the total emission rate.

To conclude, we integrated Au-SiO_2_-Si tunnel junctions into silicon photonics and showcased the polarized emission of these very broadband and geometrically and electronically tunable sources. In addition, we modeled the inelastic tunneling transition and the optical environment of our MOS system and got a good agreement regarding emission spectrum, polarization, and bias dependent emitted power. However, we see a discrepancy in emission efficiency between our model and our experiment, which we attribute primarily to nonidealities of our tunnel oxides and subsequent additional conductive channels. We note, however, that from our inelastic transition calculation, an ideal MOS structure should be very comparable in absolute power, if not better, to a MIM device. This highlights the desirability and possible benefits of engineering the inelastic transitions, e.g., with quantum wells^20^ or different semiconducting structures, and of considering the role of electron momentum for these transitions,^21^ in order to ultimately realize momentum-matched, efficient photon sources for on-chip applications.

## Supporting Information Available

Additional information about fabrication, cutback measurement, emission measurement setup and calibration, 1D mode enhancement, transverse multimodes, cavity model, tunneling calculations, device performance spread, emission at different bias voltages, and electrical characteristics of the tunnel junctions (PDF)

## References

[ref1] Lambe J.; McCarthy S. L. Light Emission from Inelastic Electron Tunneling. Phys. Rev. Lett. 1976, 37, 923–925. 10.1103/PhysRevLett.37.923.

[ref2] Parzefall M.; et al. Antenna-coupled photon emission from hexagonal boron nitride tunnel junctions. Nat. Nanotechnol. 2015, 10, 1058–1063. 10.1038/nnano.2015.203. 26367108

[ref3] Qian H.; Hsu S.-W.; Gurunatha K.; Riley C. T.; Zhao J.; Lu D.; Tao A. R.; Liu Z. Efficient light generation from enhanced inelastic electron tunnelling. Nat. Photonics 2018, 12, 485–488. 10.1038/s41566-018-0216-2.

[ref4] Yu A.; et al. Visualization of Nanoplasmonic Coupling to Molecular Orbital in Light Emission Induced by Tunneling Electrons. Nano Lett. 2018, 18, 3076–3080. 10.1021/acs.nanolett.8b00613. 29660286

[ref5] Cassidy M. C.; et al. Demonstration of an ac Josephson junction laser. Science 2017, 355, 939–942. 10.1126/science.aah6640. 28254938

[ref6] Landsman A. S.; et al. Ultrafast resolution of tunneling delay time. Optica, OPTICA 2014, 1, 343–349. 10.1364/OPTICA.1.000343.

[ref7] Cazier N.; et al. Electrical excitation of waveguided surface plasmons by a light-emitting tunneling optical gap antenna. Opt. Express 2016, 24, 3873 10.1364/OE.24.003873. 26907040

[ref8] Berndt R.; Gimzewski J. K.; Johansson P. Inelastic tunneling excitation of tip-induced plasmon modes on noble-metal surfaces. Phys. Rev. Lett. 1991, 67, 3796–3799. 10.1103/PhysRevLett.67.3796. 10044828

[ref9] Leon C. C.; Rosławska A.; Grewal A.; Gunnarsson O.; Kuhnke K.; Kern K.; et al. Photon superbunching from a generic tunnel junction. Sci. Adv. 2019, 5, eaav4986 10.1126/sciadv.aav4986. 31093525 PMC6510551

[ref10] Fung E.-D.; Venkataraman L. Too Cool for Blackbody Radiation: Overbias Photon Emission in Ambient STM Due to Multielectron Processes. Nano Lett. 2020, 20, 8912–8918. 10.1021/acs.nanolett.0c03994. 33206534

[ref11] Parzefall M.; Novotny L. Light at the End of the Tunnel. ACS Photonics 2018, 5, 4195–4202. 10.1021/acsphotonics.8b00726.

[ref12] Namgung S.; et al. Ultrasmall Plasmonic Single Nanoparticle Light Source Driven by a Graphene Tunnel Junction. ACS Nano 2018, 12, 2780–2788. 10.1021/acsnano.7b09163. 29498820

[ref13] Zhu Y.; Cui L.; Abbasi M.; Natelson D. Tuning Light Emission Crossovers in Atomic-Scale Aluminum Plasmonic Tunnel Junctions. Nano Lett. 2022, 22, 8068 10.1021/acs.nanolett.2c02013. 36197739

[ref14] Zhang C.; Hugonin J.-P.; Coutrot A.-L.; Sauvan C.; Marquier F.; Greffet J.-J.; et al. Antenna surface plasmon emission by inelastic tunneling. Nat. Commun. 2019, 10, 4949 10.1038/s41467-019-12866-3. 31666511 PMC6821910

[ref15] Watanabe J.; Uehara Y.; Murota J.; Ushioda S. Light Emission from Si-Metal-Oxide-Semiconductor Tunnel Junctions. Jpn. J. Appl. Phys. 1993, 32, 99 10.1143/JJAP.32.99.

[ref16] Uehara Y.; Watanabe J.; Fujikawa S.; Ushioda S. Light-emission mechanism of Si-MOS tunnel junctions. Phys. Rev. B 1995, 51, 2229–2238. 10.1103/PhysRevB.51.2229. 9978972

[ref17] Cartier E.; Tsang J. C.; Fischetti M. V.; Buchanan D. A. Light emission during direct and Fowler-Nordheim tunneling in ultra thin MOS tunnel junctions. Microelectron. Eng. 1997, 36, 103–106. 10.1016/S0167-9317(97)00025-7.

[ref18] Wang F.; et al. Silicon-Based Quantum Mechanical Tunnel Junction for Plasmon Excitation from Low-Energy Electron Tunneling. ACS Photonics 2021, 8, 1951–1960. 10.1021/acsphotonics.0c01913.

[ref19] Shalem G.; Erez-Cohen O.; Mahalu D.; Bar-Joseph I. Light Emission in Metal–Semiconductor Tunnel Junctions: Direct Evidence for Electron Heating by Plasmon Decay. Nano Lett. 2021, 21, 1282–1287. 10.1021/acs.nanolett.0c03945. 33497237 PMC7883388

[ref20] Qian H.; Li S.; Hsu S.-W.; Chen C.-F.; Tian F.; Tao A. R.; Liu Z.; et al. Highly-efficient electrically-driven localized surface plasmon source enabled by resonant inelastic electron tunneling. Nat. Commun. 2021, 12, 3111 10.1038/s41467-021-23512-2. 34035272 PMC8149681

[ref21] Kuzmina A.; et al. Resonant Light Emission from Graphene/Hexagonal Boron Nitride/Graphene Tunnel Junctions. Nano Lett. 2021, 21, 8332–8339. 10.1021/acs.nanolett.1c02913. 34607425

[ref22] Liu L.; et al. Waveguide-Integrated Light-Emitting Metal–Insulator–Graphene Tunnel Junctions. Nano Lett. 2023, 23, 3731–3738. 10.1021/acs.nanolett.2c04975. 37097286 PMC10176563

[ref23] Martín-Jiménez A.; et al. Electronic Temperature and Two-Electron Processes in Overbias Plasmonic Emission from Tunnel Junctions. Nano Lett. 2021, 21, 7086–7092. 10.1021/acs.nanolett.1c00951. 34152778

[ref24] Khurgin J. B. How to deal with the loss in plasmonics and metamaterials. Nat. Nanotechnol. 2015, 10, 2 10.1038/nnano.2014.310. 25559961

[ref25] Parzefall M.; Bharadwaj P.; Novotny L. Antenna-Coupled Tunnel Junctions. In Quantum Plasmonics; Bozhevolnyi S. I., Martin-Moreno L., Garcia-Vidal F., Eds.; Springer International Publishing: 2017; pp 211–236; 10.1007/978-3-319-45820-5_10.

[ref26] Bardeen J. Tunnelling from a Many-Particle Point of View. Phys. Rev. Lett. 1961, 6, 57–59. 10.1103/PhysRevLett.6.57.

[ref27] Reittu H. J. Fermi’s golden rule and Bardeen’s tunneling theory. American Journal of Physics 1995, 63, 940–944. 10.1119/1.18037.

[ref28] Drexhage K. H. Influence of a dielectric interface on fluorescence decay time. J. Lumin. 1970, 1–2, 693–701. 10.1016/0022-2313(70)90082-7.

[ref29] Snoeks E.; Lagendijk A.; Polman A. Measuring and Modifying the Spontaneous Emission Rate of Erbium near an Interface. Phys. Rev. Lett. 1995, 74, 2459–2462. 10.1103/PhysRevLett.74.2459. 10057933

[ref30] Kuhn H. Classical Aspects of Energy Transfer in Molecular Systems. J. Chem. Phys. 1970, 53, 101–108. 10.1063/1.1673749.

[ref31] Novotny L.; Hecht B. Principles of Nano-Optics; Cambridge University Press: 2012.

[ref32] Cheng B.; Zellweger T.; Malchow K.; Zhang X.; Lewerenz M.; Passerini E.; Aeschlimann J.; Koch U.; Luisier M.; Emboras A.; Bouhelier A.; Leuthold J.; et al. Atomic scale memristive photon source. Light Sci. Appl. 2022, 11, 78 10.1038/s41377-022-00766-z. 35351848 PMC8964763

[ref33] Green M. A.; Zhao J.; Wang A.; Reece P. J.; Gal M. Efficient silicon light-emitting diodes. Nature 2001, 412, 805–808. 10.1038/35090539. 11518962

[ref34] Chang S. T.; et al. The band-edge light emission from the metal-oxide-silicon tunneling diode on (110) substrates. Solid-State Electron. 2002, 46, 1113–1116. 10.1016/S0038-1101(02)00051-5.

[ref35] Lee S.-H.; Cho B.-J.; Kim J.-C.; Choi S.-H. Quasi-breakdown of ultrathin gate oxide under high field stress. Proceedings of 1994 IEEE International Electron Devices Meeting 1994, 605–608. 10.1109/IEDM.1994.383337.

[ref36] Depas M.; Nigam T.; Heyns M. M. Soft breakdown of ultra-thin gate oxide layers. IEEE Trans. Electron Devices 1996, 43, 1499–1504. 10.1109/16.535341.

