# Supplementary material for: Broadband Tunable Infrared Light Emission from Metal-Oxide-Semiconductor Tunnel Junctions in Silicon Photonics
Source: Nano Lett. 2023 Dec 5;24(3):859–65. doi: 10.1021/acs.nanolett.3c03684 (PMC10811661; doi:10.1021/acs.nanolett.3c03684)
Supplement: Supplementary file 1 — nl3c03684_si_001.pdf [file nl3c03684_si_001.pdf]

# Broadband Tunable Infrared Light Emission from Metal-Oxide-Semiconductor Tunnel Junctions in Silicon Photonics

**Michael Doderer[1], Killian Keller[1], Joel Winiger[1], Michael Baumann[1], Andreas Messner[1], David Moor[1], Daniel Chelladurai[1], Yuriy Fedoryshyn[1], Juerg Leuthold[1], Jared Strait[2], Amit Agrawal[2], Henri J. Lezec[2], Christian Haffner[3]**

*1. Institute of Electromagnetic Fields (IEF), ETH Zurich, 8092 Zurich, Switzerland*

*2. Physical Measurement Laboratory, National Institute of Standards and Technology, Gaithersburg, MD 20899, USA*

*3. Interuniversity Microelectronics Centre (imec), Remisebosweg 1, 3001 Leuven, Belgium*

*Corresponding authors: michael.doderer@ief.ee.ethz.ch, christian.haffner@imec.be*

# Supplementary information

## Fabrication

We fabricated the tunnel devices from a $\approx$ 340 nm thick silicon-on-insulator (SOI), which in a first step were globally doped to approximately $n_{\text{phosphorus}} \approx (5 \cdot 10^{18})\ \text{cm}^{-3}$ via a two-step ion implantation and activation and diffusion annealing. Then, using e-beam lithography, the silicon was fully etched with an inductively coupled plasma etch (ICP) to electrically separate the different devices and to form the photonic waveguides. A following partial etch formed the thin ridges for the silicon contact. Next, using optical lithography resist and a lift-off, nickel was deposited via electron beam evaporation onto the silicon and subsequently annealed in forming gas to form a silicide contact. After that, a conformal $SiO_2$ insulating layer was deposited using plasma-enhanced atomic layer deposition (PE-ALD), which was then locally opened down to the silicon using electron-beam (e-beam) lithography and reactive ion etching (RIE), in order to define the tunnel junction areas. A very short (2 s) dip in buffered hydrofluoric acid (1:7 solution) was then used to remove the residual oxide over the tunnel area (ALD and native oxide) before the samples were directly transferred to the PE-ALD to deposit the tunnel barriers. Since an oxygen plasma was used in the PE-ALD which oxidizes the silicon surface, there is always an offset of tunnel oxide thickness, as illustrated in Supplement Figure 1. The deposited thicknesses were measured via ellipsometry on a pure silicon reference sample, and the final thickness used was $d_{\text{SiO2}} = 2\ \text{nm}$. The last step was then the e-beam lithography and e-beam evaporation of $\approx$ 1 nm Titanium and $\approx$ 100 nm Gold to define the second electrode.

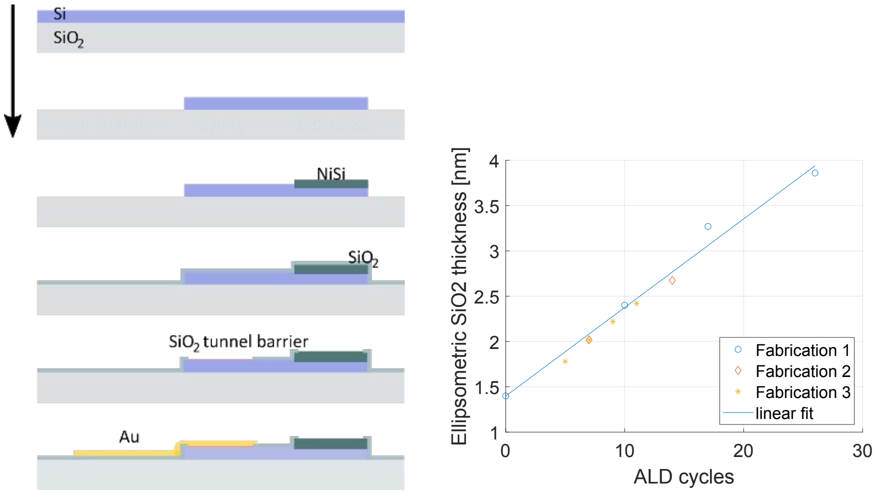


*Supplement Figure 1: left: The fabrication steps are illustrated. right: Ellipsometrically measured tunnel oxide thickness on silicon, dependent on cycle numbers in the PE-ALD at 200 °C. With the use of an oxygen plasma in the growth, there is always some offset of tunnel oxide thickness, i.e. the tunnel oxide consists of both plasma-grown and ALD-grown oxide.*

## Cutback Measurement

To characterize the propagation losses of the hybrid plasmonic mode, we performed a cutback measurement, where several devices with different hybrid propagation lengths were measured. After setup and grating coupler loss subtraction, the resulting transmission losses are averaged from 1480 nm to 1560 nm to minimize resonant effects. In addition to the propagation length, the coupling efficiency between the photonic and hybrid WG can also be extracted as $\eta_{coupling} = 74^{+6}_{-8}\,\%$, as the intersection of the fitted line at zero hybrid length corresponds to twice the coupling loss.

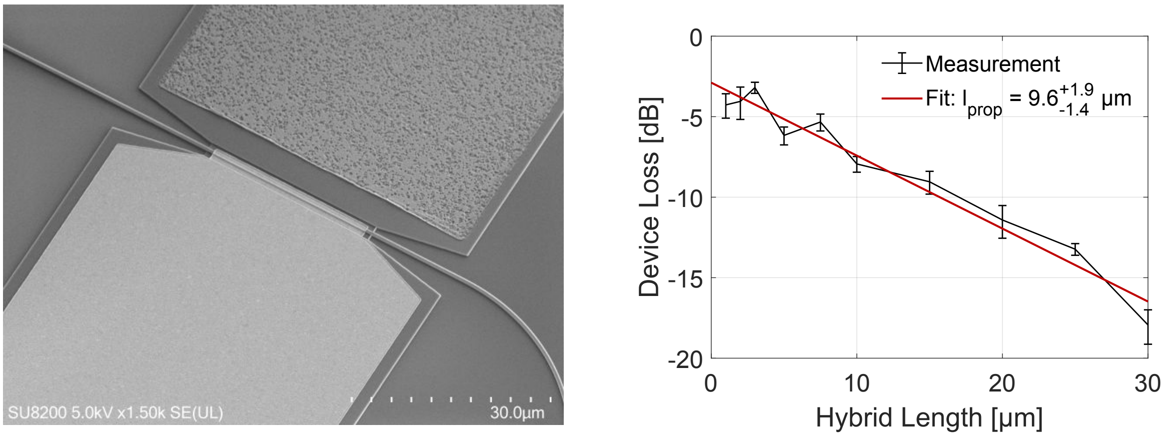


*Supplement Figure 2: left: an SEM image of the cutback measurement structure is shown. right: the extracted device losses for various hybrid mode section lengths are shown, including the linear fit in the semi logarithmic plot to extract the losses. The loss uncertainties are taken from the uncertainty of the fit and signify the 95% confidence bounds.*

## Emission Measurement Setup and Calibration

The emission is coupled to a silicon photonic waveguide and guided to the edge of the diced and mechanically polished chip, where it is collected by a high numerical aperture (NA) NIR microscope objective (50x, NA=0.65). A c-coated linear polarizer filters the emission and an anti-reflective coated objective lens then focuses the light to a Czerny-Turner spectrometer with a 45 lines per millimeter, 1.75 µm blazed grating. There, a thermoelectrically cooled InGaAs focal plane array is used for alignment and power measurements, and a liquid nitrogen cooled linear InGaAs detector is used to record the spectra.

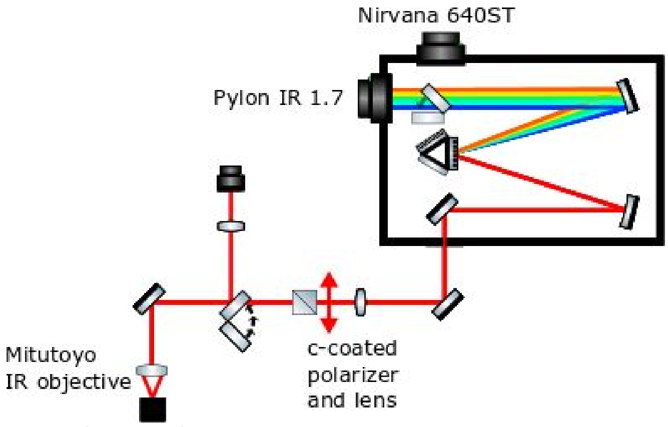


*Supplement Figure 3: Sketch of the used emission measurement setup, where either the Nirvana or Pylon detector was used for power or spectral measurements respectively.*

To measure the emitted power via the InGaAs focal plane array, the polished edge facet is focused on to the detector with a mirror installed in the spectrometer's grating turret. Each measurement takes one emission image and one background image for dark subtraction. The recorded pixel-intensities are summed up over a limited area where the emission is recorded. This summed intensity corresponds to the total number of photons being detected due to the detector's unity gain. The same is done on a second area on the signal image, right below the emitting area. From this second dark area, the standard deviation of the pixel values is calculated to estimate the dark current noise in the measurement, which is shown in the error bars in the main text.

To estimate the actual emitted power into the waveguide as well as the source efficiency, the following values for the losses are considered, where the dipole-photon coupling factor is only used in the efficiency estimation.

| Efficiency factor | Value | Comments |
|---|---|---|
| **Dipole excitation to photon** | TM: 0.02<br>TE: 0.018 | From 3D FDTD cavity simulations with a dipole source; average over the InGaAs detection window (1.1 µm to 1.65 µm).<br>Verified with separate 3D FEM simulations. |
| **WG loss** | 0.15 | Approximate NIR propagation loss trough 650 um Si WG doped to 5 x $10^{18}$ [1];<br>Verified by photonic cutback measurement |
| **WG edge coupling transmission** | 0.69 | Fresnel reflection coefficient Si-Air.<br>Verified by 3D FDTD |

| | | |
|---|---|---|
| **Collection efficiency** | 0.64 | Collected light under the Gaussian beam approximation with collection NA = 0.65 |
| **Objective transmission** | 0.6 | Approximate transmission through the objective lens in the NIR, taken from data sheet |
| **Mirrors & lenses loss** | 0.965 | |
| **Nirvana 640 ST quantum efficiency** | 0.86 | From datasheet |
| **Total collection efficiency Dipole to detector** | TM: 0.00066<br>TE: 0.00060 | |

The efficiency values in the main text thus consist of the vacuum source efficiency ( $\approx 10^{-6}$) times the transversal, i.e. heterostructure, LDOS enhancement factor ( $\approx 300$)

For the spectral measurements, the spectrometer’s wavelength was calibrated using a Hg/Ne calibration source. The relative system response calibration was performed with a known Halogen lamp, which was beforehand measured on a NIST traceable NIR spectrometer.

## 1D mode enhancement

The 1-dimensional mode density enhancement is calculated by the angular spectrum representation of the dipole's emitted field. We calculate the total field at the dipoles position by applying the appropriate boundary conditions from the layer stacks permittivities[2]. The method is based upon and explained in detail in work from Parzefall et. al.[3] We calculate the optical mode density enhancement of the heterostructure via:

$$\frac{P}{P_0} = \frac{\rho_{\text{opt}}}{\rho_0} = \frac{3}{2}\int_0^{\infty} Re\left(\frac{\left(\frac{k_{||}}{k_0}\right)^3}{\frac{k_{zi}}{k_0}\varepsilon_i}\left(1 + c_i^{\uparrow} + c_i^{\downarrow}\right)\right) ds$$

where $k_0$ is the vacuum wavevector, $k_{||}$ is the in plane wavevector component, and $k_{zi}$ is the out of plane wavevector component in material $i$ (i.e. $SiO_2$). $c_i^{\uparrow}$ and $c_i^{\downarrow}$ are the upwards and downwards traveling complex e-field amplitudes, and $\varepsilon_i$ is the permittivity of material $i$.

Applied to our MOS structure, we can calculate the dipole emission enhancement as shown in Supplement Figure 4, where the TM0 and TM1 modes are visible as resonances. The integrated area under the TMx mode then corresponds to the value of $\rho_t/\rho_0$ in the main text, which is mentioned to be around $25$ % of the total emission.

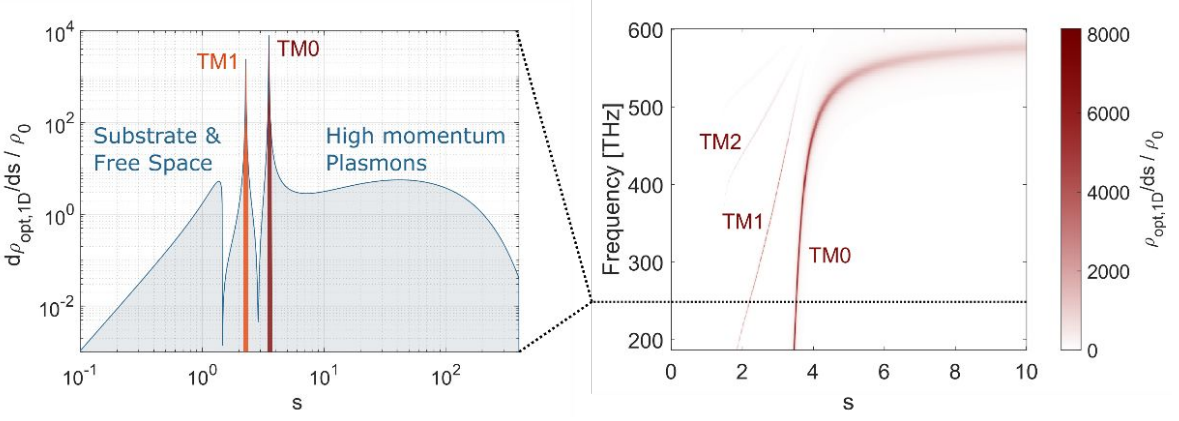


*Supplement Figure 4: Calculated dipole radiation enhancement purely due to the MOS heterostructure. The normalized wavenumber $s = k_{||}/k_0$ is used, and replicates the $n_{eff}$ of the supported modes. The strong plasmonic resonance for frequencies above 550 THz is clearly visible as a broadening of the TM0 mode's resonance and its increase in wavenumber.*

### Influence of Silicon Thickness

While the tunnel oxide thickness is very limited in variability to maintain direct tunnelling, we could potentially vary the thickness of the silicon device layer. Supplement Figure 5 shows the calculations of the emission efficiency into the fundamental TM0 mode as well as the total emission into the TM0 for a heterostructure with varying silicon thickness.

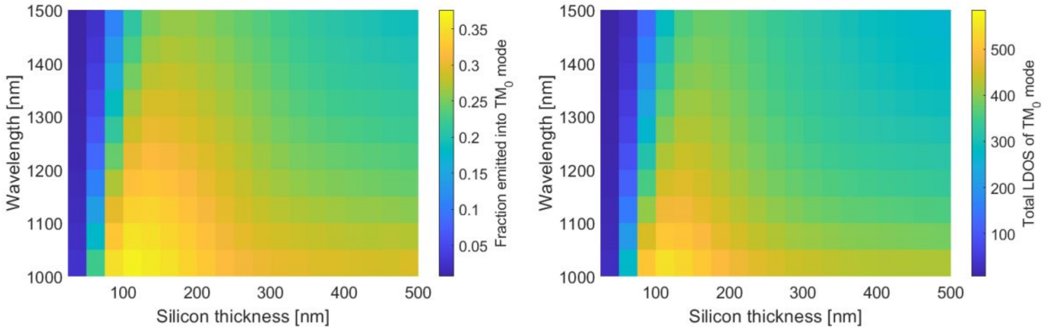


*Supplement Figure 5: Left: For the heterostructure Au-SiO2-Si the emission efficiency into the TM0 mode is plotted for varying silicon thickness and wavelengths. Right: For the same structure and variations, the total emission into the TM0 is plotted, instead of the efficiency as in the left plot.*

## Transverse multimodes

To better understand the influence of the width of the tunnel section, we perform 3D FEM simulations with a single dipole emitter in the center of the thickness of the oxide of a hybrid waveguide at 1400 nm. The resulting optical field in the hybrid mode WG is overlapped with the guided eigenmodes, showcasing the excitation of the different modes for varying waveguide widths. The overall emitted power for a single dipole emitter does not change, however in the transverse multi-mode case, the emitted power is distributed relatively evenly between the different modes.

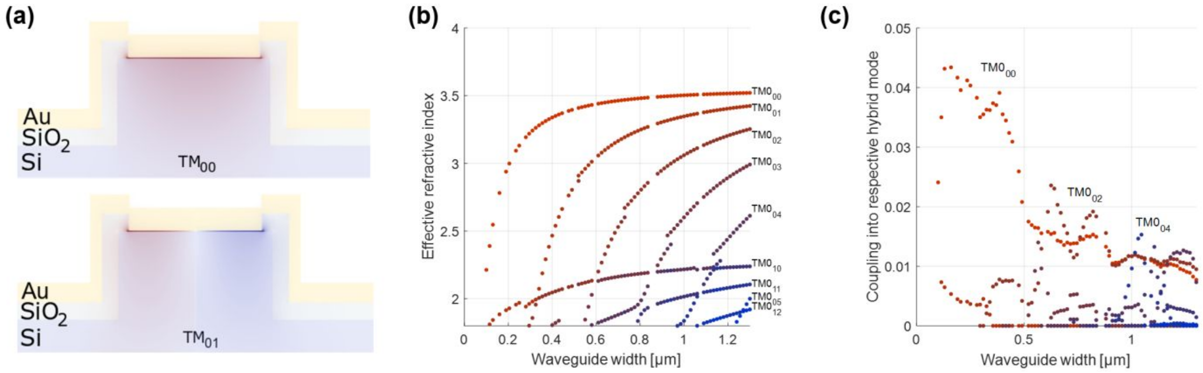


*Supplement Figure 6: a: The transverse fundamental mode TM00 and the first order mode TM01 are illustrated. b: Resulting from 2D eigenmode analysis, the real part of the effective mode indices at vacuum wavelength of 1400 nm are plotted versus an increasing waveguide width. c: Originating from a central dipole excitation, coupling into the various modes is shown for varying WG width.*

The coupling into the respective mode is calculated as

$$\eta_{coupling,Dipole-mode} = \chi_{field_{dipole},WG_{mode}} \cdot \frac{\iint_{field_{dipole}} \sqrt{P_x^2 + P_y^2 + P_z^2}\, dA}{\iint_{AllDomainbounds} \sqrt{P_x^2 + P_y^2 + P_z^2}\, dA}$$

Where $\chi$ is the field overlap and $P_{x,y,z}$ the Poynting vector component along x, y, or z.

As is evident in Supplement Figure 6b, with increasing width the number of transverse modes increases very quickly. As a result, a wide (25 μm), highly transversally multi-moded LETJ can support around 120 different modes, all with effective refractive indices between 1.5 and 3.5. In addition, the lateral dipole position influences which modes can be excited, as

illustrated in Supplement Figure 6c, where only the even transverse modes are excited due to the central position of the simulatied excitation-dipole.

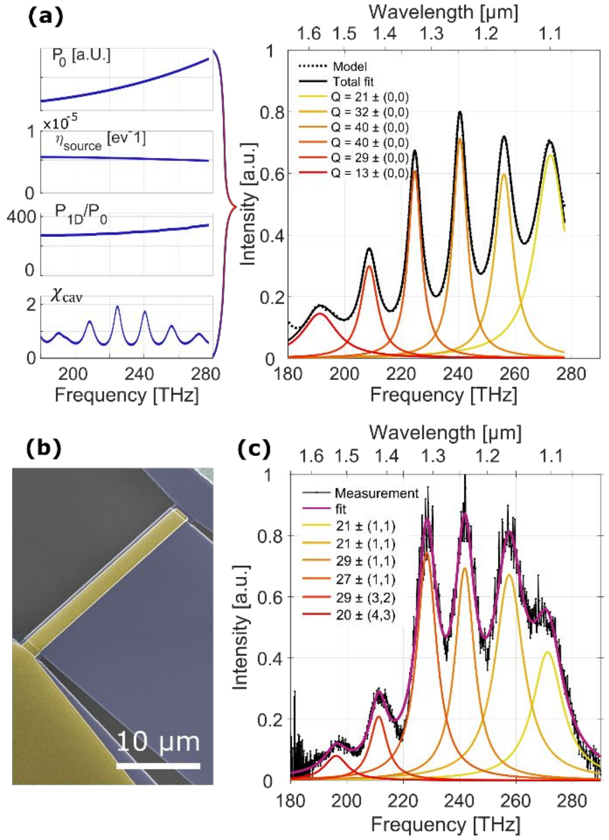


*Supplement Figure 7: (a): Identical sub-figure from the main text, showcasing the different contributions to the simulated emission spectrum on the right. (b): colorized SEM image of wide, transversally multimoded device, whose TM spectrum is shown in (c). The same longitudinal cavity modes as for the single-moded device can be observed from the emission under a bias voltage* $V_b = 2.5\ V$ $(I_s \approx (7.04 \pm 0.08)^{\mu A}/_{\mu m^2})$.

The effect of non-homogenous tunneling path distribution should be more pronounced the smaller the cavity area becomes, as fewer and fewer tunnel paths will contribute to the emission. Reinforcing this hypothesis is the recorded spectrum of a transversally very large tunnel device, illustrated in Supplement Figure 7. There, we assume that in total more tunnel paths are actively emitting and thus effectively more excitation dipoles are distributed inside the cavity. This leads to a better agreement in the cavity mode intensities with the simulated model where evenly distributed dipoles are assumed. However, the wide waveguide structure now supports more than 120 transversally higher order modes, all of which will be excited. As we only collect a significant amount of light from the first few modes, all having similar effective refractive indices – and thus similar longitudinal resonance frequencies – we still record the cavity resonances, albeit with reduced quality factors due to higher order mode averaging.

## Cavity Model

To accurately model the resonances of the measured emission spectrum, we consider the emitter inside a Fabry-Pérot cavity with frequency-dependent mode index, loss, and reflectivities.

We assume that the inelastic tunneling processes occur incoherently and can be regarded as spontaneously emitting dipole sources distributed along the cavity's length $L$. The spontaneous emission enhancement due to the increased density of optical states can be calculated with the field enhancement at the location of the dipole.[4] As we have many dipole-like emitters throughout the cavity, we need to average the field enhancement inside the cavity over the entire length to model the total emission.[5]

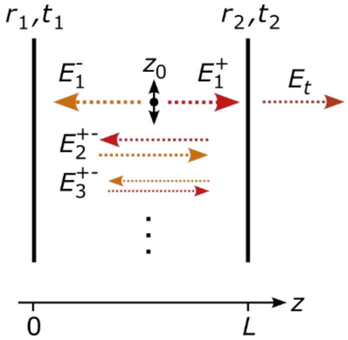

*Supplement Figure 8: Sketch of the Fabry-Pérot cavity model used.*

Inside the cavity, we have a hybrid plasmonic mode with an effective refractive index $n_{\text{eff}}$ and resulting k-vector $k = k' + ik'' = \frac{\omega}{c_0} real(n_{\text{eff}}) + i \cdot \frac{\omega}{c_0} imag(n_{\text{eff}})$, where $\omega$ is the radial frequency and $c_0$ the vacuum speed of light. The emitted field $E_{\text{t}}$ leaving the cavity can be calculated as the sum of all the fields inside, originating from a dipole at position $z_0$. To get the total emission, we integrate the field enhancement over all different dipole positions $0 < z_o < L$.

We define a propagator $\chi(z) = e^{-ik'z} e^{-k''z}$ and the mirror reflection and transmission coefficients $r_{1,2}$ and $t_{1,2}$ respectively. Forming a geometric series of the reflected fields inside the cavity, we can define the coefficient $H$

$$H(\omega) = \frac{1}{1 - r_1 r_2 \cdot \chi(2L)}$$

And considering the propagation from $z_0$, we can write out the emitted field as

$$E_{\text{t}}(z) = t_2 \left( H\chi(L - z_0) E_1^+ + H r_1 \chi(z_0 + L) E_1^- \right)$$

The total field enhancement than becomes:

$$\chi_{\text{cav}} = \frac{1}{L} \int_0^L \frac{\left|\overrightarrow{E_t}\right|^2}{\left|\vec{E}_0\right|^2} dz = \frac{1}{L} \int_0^L H t_2 \left( \chi(L - z_0) + r_1 \chi(z_0 + L) \right) dz_0$$

Since the reflectors are strongly frequency dependent, they are simulated with 3D FDTD, where the geometry is modelled as close to the fabricated device as possible.

## Tunneling calculations

Both elastic and inelastic tunneling are treated as a first order perturbation on the Hamiltonian of the electrode/barrier system[6]. This assumption is valid as long as the electrons in both electrodes are only weakly coupled, which is the case for the barrier thicknesses considered in this work[7,8]. The total system Hamiltonian can thus be written as[2,9]

$$\hat{H} = \hat{H}_0 + \hat{H}_{\text{el}} + \hat{H}_{\text{inel}}$$

Where we have $\hat{H}_{el}$ and $\hat{H}_{inel}$ as the elastic and inelastic perturbative terms.

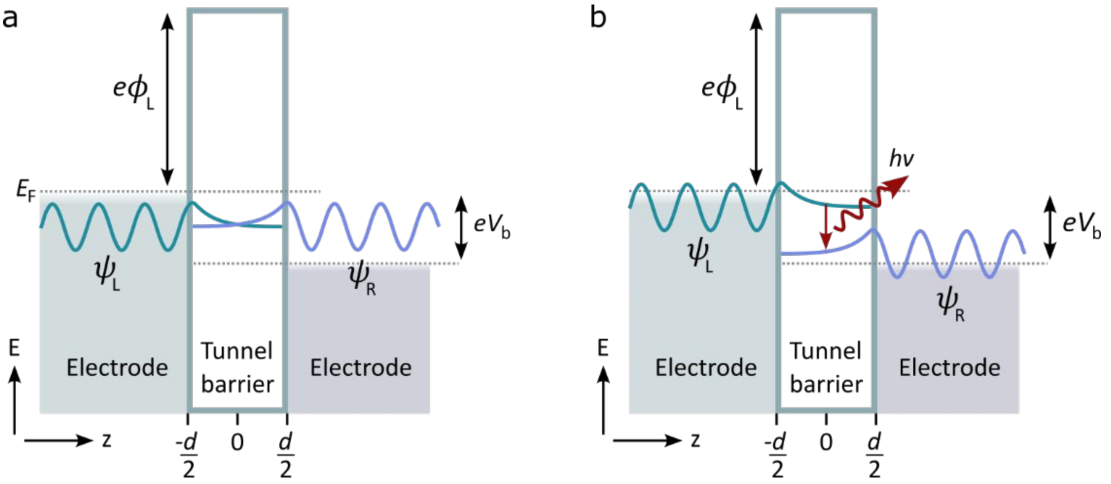


*Supplement Figure 9: left: the case of the elastic tunneling rate calculation is sketched. Right: the inelastic tunneling rate calculation considers all transition where the electrons in the left and right electrode have energies differing by $h\nu$.*

We now want to determine the wavefunctions $\psi_{\text{L,R}}$ separately for both electrodes.

### Elastic Tunneling Rate

Due to the weak coupling, we model each electrode's potential separately:

$$V_{\text{L}} = \begin{cases} V_0, & z < -\frac{d}{2} \\ V_{\text{barrier}}, & z \geq -\frac{d}{2} \end{cases}, V_{\text{R}} = \begin{cases} V_0, & z > \frac{d}{2} \\ V_{\text{barrier}}, & z \leq \frac{d}{2} \end{cases}$$

Where $d$ is the barrier thickness. As a simplification, we assume the barrier to be rectangular. A more detailed calculation could be made with a discretization of the barrier potential[10], which would however require a fully numerical calculation of the transmission rates.

With rectangular potentials, the electrons' wavenumbers become

$$k_{\text{L}}' = \sqrt{\frac{2m_{\text{b}}E}{\hbar}},\ k_{\text{L}}'' = \sqrt{\frac{2m_{\text{b}}(E - e\phi_{\text{L}})}{\hbar}}$$

Where $k_{\text{L}}'$ and $k_{\text{L}}''$ are the electron's wavenumber in the left electrode and the left barrier respectively. $m_{\text{b}}$ is the effective electron mass inside the barrier, which for $SiO_2$ is assumed as[11] $m_{\text{b,SiO2,parabolic}} = 0.3\, m_0$. $\phi_{\text{L}}$ is the left electrode's barrier height. We apply the bias voltage $V_{\text{b}}$ on the right electrode and get for its wavenumber with the assumed rectangular barrier

$$k_{\text{R}}' = \sqrt{\frac{2m_{\text{b}}(E - eV_{\text{b}})}{\hbar}},\ k_{\text{R}}'' = \sqrt{\frac{2m_{\text{b}}(E - e\phi_R)}{\hbar}}$$

Following Bardeen's tunneling theory[6,7], we get the elastic transition matrix element $T(E)$

$$T(E) = \frac{\hbar^2}{2m_\mathrm{b}}\left(\psi_\mathrm{L}\frac{d\psi_\mathrm{R}^*}{dz} - \psi_\mathrm{R}^*\frac{d\psi_\mathrm{L}}{dz}\right) \tag{1}$$

Where $\psi_\mathrm{L,R}$ are the unperturbed wavefunctions on the left and right side ($d\rightarrow\infty$).

For our rectangular barrier system, we can solve the time-independent Schrödinger equation to get the following wavefunctions

$$\psi_\mathrm{L} = \frac{2k'_\mathrm{L}}{k'_\mathrm{L} + ik''_\mathrm{L}}\mathrm{e}^{-k''_\mathrm{L}\left(z+\frac{d}{2}\right)},\ \psi_\mathrm{R} = \frac{2k'_\mathrm{R}}{k'_\mathrm{R} + ik''_\mathrm{R}}\mathrm{e}^{-k''_\mathrm{R}\left(-\frac{d}{2}-z\right)} \tag{2}$$

Inserting (**Error! Reference source not found.**) into (**Error! Reference source not found.**) leaves us with the elastic tunnelling transition matrix element for a rectangular barrier

$$T(E) = \frac{\hbar^2}{m_\mathrm{b}} \cdot \frac{2\left(k''_\mathrm{L} + k''_\mathrm{R}\right) \cdot k'_\mathrm{L}k'_\mathrm{R}}{\left(k''_\mathrm{L} + ik'_\mathrm{L}\right)\left(k''_\mathrm{R} + ik'_\mathrm{R}\right)} \cdot \mathrm{e}^{-d \cdot \frac{k''_\mathrm{L} + k''_\mathrm{R}}{2}}$$

By integrating over all energies and including the electrodes, respective density of states $\rho_\mathrm{L/R}$ , we end up with the total elastic electron transition rate $\Gamma_\mathrm{el}$.

$$\Gamma_\mathrm{el} = \frac{2\pi}{\hbar}\int_0^{eV_\mathrm{b}} |T(E)|^2\rho_\mathrm{L}(E)\rho_\mathrm{R}(E)dE$$

**Inelastic tunnelling rate**

For our considered case of weak coupling, we can treat the inelastic tunnelling transition in the same way as the elastic transition as a perturbation to the system Hamiltonian. Here we use the Light-Matter interaction Hamiltonian

$$\hat{H}_{inel} = -\frac{q}{m}\hat{\boldsymbol{A}} \cdot \hat{\boldsymbol{p}}$$

With $\hat{\boldsymbol{A}}$ as the vector potential operator and $\hat{\boldsymbol{p}} = -i\hbar\, d/dz$ as the momentum operator. The inelastic transition matrix element $P(E,h\nu)$ is the expectation value when applying the momentum operator:

$$P(E,h\nu) = \langle\psi_\mathrm{R}|\vec{p}_\mathrm{z}|\psi_\mathrm{L}\rangle = -i\hbar\int_{-d/2}^{d/2}\left[\psi_\mathrm{R}^*\frac{\mathrm{d}}{\mathrm{dz}}\psi_\mathrm{L}\right]dz$$

We assume that the vector potential inside the rectangular tunnel barrier is varying much slower than the electron wavefunction, which allows us to evaluate this transition matrix element at any point inside the barrier. This gives us the evaluated element

$$P(E,h\nu) = \frac{8i\hbar k'_\mathrm{L}k'_\mathrm{R}k''_\mathrm{L}}{\left(k'_\mathrm{L} + ik''_\mathrm{L}\right)\left(k'_\mathrm{R} - ik''_\mathrm{R}\right)} \cdot e^{-d\frac{k''_\mathrm{L} + k''_\mathrm{R}}{2}} \cdot \frac{\sinh\left(\frac{d}{2}\left(k''_\mathrm{L} - k''_\mathrm{R}\right)\right)}{k''_L - k''_R}$$

Following Fermi's golden rule we can then calculate the total spectral inelastic tunneling rate by integrating over all allowed energies for the photon energy $h\nu$ [9]

$$\gamma_\mathrm{inel}(h\nu) = \frac{e^2}{6\nu m_\mathrm{b}^2\varepsilon_0} \cdot \rho_\mathrm{opt}\int_{h\nu}^{eV_\mathrm{b}} |P(E)|^2 \cdot \rho_L(E) \cdot \rho_\mathrm{R}(E - h\nu)dE$$

$\varepsilon_0$ is the vacuum permittivity, $e$ the electron charge

We assume the gold to have a constant electronic density of states (eDOS) over the considered electron energies[3], while we take a square-root dependence of the eDOS at the band-edges of silicon due to the parabolic dispersion of electrons.

Parameters for the tunnel rate calculations:

| Symbol | Value | Description |
|---|---|---|
| $\boldsymbol{\phi}_{\mathbf{L}}$ | $3\ eV$ | Barrier height left electrode |
| $\boldsymbol{\phi}_{\mathbf{R}}$ | $4.3\ eV$ | Barrier height right electrode |
| $\boldsymbol{E}_{\mathbf{F,Au}}$ | $5.53\ eV$ | Fermi energy in gold |
| | | |
| $\boldsymbol{\chi}_{\mathbf{Si}}$ | $4.1\ eV$ | Electron affinity silicon |
| $\boldsymbol{E}_{\mathbf{Si,Bg}}$ | $1.12\ eV$ | Silicon Bandgap energy |
| $\boldsymbol{m}_{\mathbf{Si,c}}$ | $1.08\ m_0$ | Conduction electron effective mass |
| $\boldsymbol{m}_{\mathbf{Si,v}}$ | $0.56\ m_0$ | Valence electron effective mass |
| $\boldsymbol{m}_{\mathbf{barrier}}$ | $0.3\ m_0$ | Effective electron mass inside SiOx barrier[11] |

## Device performance spread

Even though the tunnel junctions were fabricated with PE-ALD, we observed quite a large spread in device performance for identical devices from the same fabrication. An example of the variation in tunnel currents at 1 V bias over the sample area of 5.5 mm x 12.8 mm is shown in Supplement Figure 10, were identical, large tunnel junctions (25 μm x 25 μm) vary in current by a factor of up to 7.

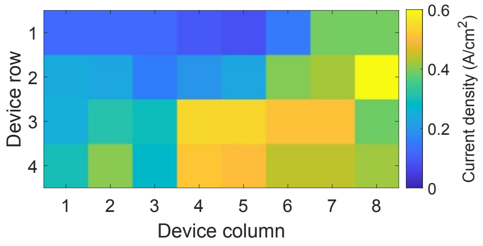


*Supplement Figure 10: Tunnel current densities for identical tunnel junctions over the area of the fabricated chip, measured at 1 V.*

We believe that this strong variability in tunnel oxide quality also leads to our large variability in observed emission efficiencies, showcased in Supplement Figure 11.

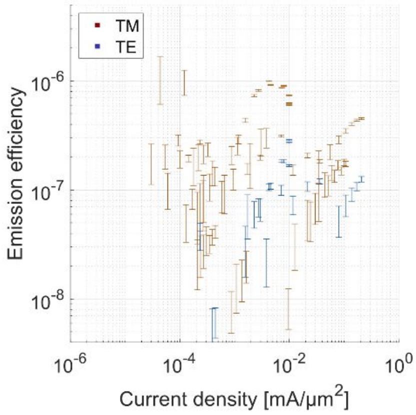


*Supplement Figure 11: Observed emission efficiencies for TM and TE emission for various current densities.*

## Emission at different bias voltages

When measuring the TM tunnel emission under different voltages, shown in Supplement Figure 12, no discernible shift in the spectra is visible, apart from an overall stronger emission. When looking at the ratios of the cavity peak intensities no clear trend is visible, as is expected from the flat vacuum source spectrum shown in Figure 2(a) in the main text, where for biases above 1.25 V no significant change in the vacuum source spectrum is visible in our system.

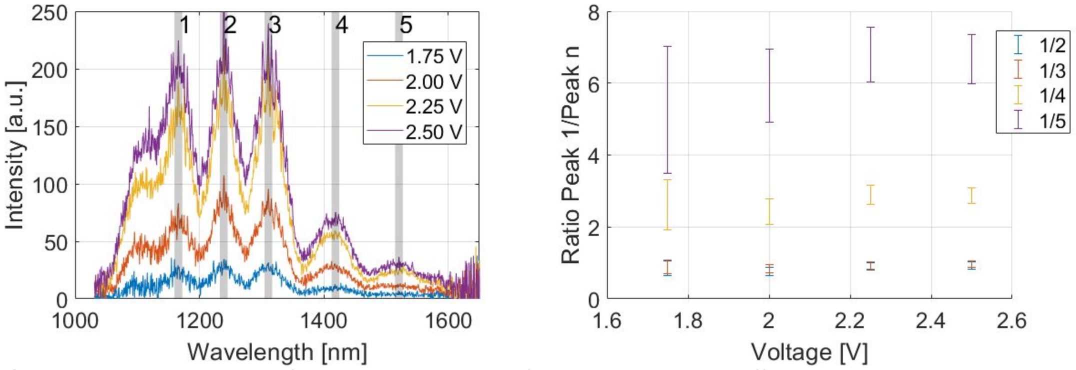


*Supplement Figure 12: Left: The TM emission of one device under different bias voltages is shown. 1-5 label the peak numbers used in the right plot, and the gray areas show the averaging windows used to calculate the peak intensity. Right: The peak intensity ratios between the peaks in the left plot are shown, with the error bars signify one standard deviation of the noise over the averaging window.*

## Electrical characteristics of the tunnel junctions

In Supplement Figure 13 an exemplary IV curve of a MOS tunnel junction is shown. We can also calculate the theoretical direct tunneling current following a Wentzel-Kramers-Brillouin approximation[12]:

$$J_{\mathrm{DT}} = \left(\frac{e^2}{2\pi h\, d_{\mathrm{SiO2}}} \cdot \left(\phi_{\mathrm{B}} - \frac{V_{\mathrm{ox}}}{2}\right) \cdot \exp\left(-4\pi\sqrt{2em_{\mathrm{e}}^*}\; d_{\mathrm{SiO2}} \cdot \sqrt{\left(\phi_{\mathrm{B}} - \frac{V_{\mathrm{ox}}}{2}\right) \cdot \frac{1}{h}}\right)\right)$$

$$- \left(\frac{e^2}{2\pi h\, d_{\mathrm{SiO2}}} \cdot \phi_{\mathrm{B}}\right)\phi_{\mathrm{B}}\exp\left(-4\pi\sqrt{2em_{\mathrm{e}}^*}\; d_{\mathrm{SiO2}}\sqrt{\phi_{\mathrm{B}}} \cdot \frac{1}{h}\right)$$

With $e$ being the electron charge, $h$ the Planck constant, $d_{\mathrm{SiO2}}$ the tunnel barrier thickness, $\phi_{\mathrm{B}}$ the tunnel barrier height, $V_{\mathrm{ox}}$ the voltage over the oxide, and $m_{\mathrm{e}}^*$ the reduced electron mass inside the tunnel barrier.

The oxide voltages for the tunneling model are calculated by solving the Poisson equation. A reasonable fit of the tunnel currents through the $2\ \mathrm{nm}$ thin oxide can be made for barrier potentials between electrode and tunnel barrier of $\phi_b = 1.45\ \mathrm{eV}$ for positive biases and $\phi_{\mathrm{b}} = 2\ \mathrm{eV}$ for negative biases. The difference in barrier potentials can be explained due to the tunneling occurring either from the silicon conduction band into the gold (positive biases) or from the gold with a large work function into the silicon (negative biases).

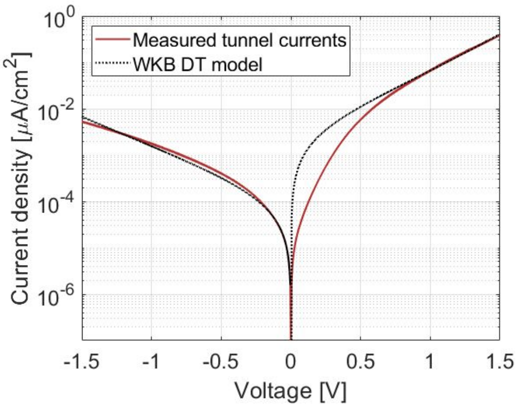

*Supplement Figure 13: Measured and calculated direct tunnel currents are shown.*

1. Soref, R. & Bennett, B. Electrooptical effects in silicon. *IEEE Journal of Quantum Electronics* **23**, 123–129 (1987).
2. Parzefall, M. & Novotny, L. Light at the End of the Tunnel. *ACS Photonics* **5**, 4195–4202 (2018).
3. Parzefall, M. *et al.* Light from van der Waals quantum tunneling devices. *Nature Communications* **10**, 292 (2019).
4. Drexhage, K. H. Influence of a dielectric interface on fluorescence decay time. *Journal of Luminescence* **1–2**, 693–701 (1970).
5. Dowling, J. P. Spontaneous emission in cavities: How much more classical can you get? *Found Phys* **23**, 895–905 (1993).
6. Bardeen, J. Tunnelling from a Many-Particle Point of View. *Phys. Rev. Lett.* **6**, 57–59 (1961).
7. Reittu, H. J. Fermi's golden rule and Bardeen's tunneling theory. *American Journal of Physics* **63**, 940–944 (1995).
8. Noguera, C. Validity of the transfer Hamiltonian approach : application to the STM spectroscopic mode. *Journal de Physique* **50**, 2587–2599 (1989).
9. Parzefall, M., Bharadwaj, P. & Novotny, L. Antenna-Coupled Tunnel Junctions. in *Quantum Plasmonics* (eds. Bozhevolnyi, S. I., Martin-Moreno, L. & Garcia-Vidal, F.) 211–236 (Springer International Publishing, 2017). doi:10.1007/978-3-319-45820-5_10.
10. Ando, Y. & Itoh, T. Calculation of transmission tunneling current across arbitrary potential barriers. *Journal of Applied Physics* **61**, 1497–1502 (1987).
11. Brar, B., Wilk, G. D. & Seabaugh, A. C. Direct extraction of the electron tunneling effective mass in ultrathin SiO2. *Appl. Phys. Lett.* **69**, 2728–2730 (1996).
12. Hirose, M. Electron tunneling through ultrathin SiO2. *Materials Science and Engineering: B* **41**, 35–38 (1996).
